# Dihydropyridine Calcium Channel Blocker Therapy and Risk of CKD Progression in Type 2 Diabetes Treated With Renin Angiotensin System Inhibitors and SGLT2 Inhibitors: A Real-World Retrospective Cohort Study

**DOI:** 10.1016/j.xkme.2026.101394

**Published:** 2026-05-08

**Authors:** Timna Agur, Tali Steinmetz, Shira Goldman, Boris Zingerman, Dana Bielopolski, Eviatar Nesher, Ittai Fattal, Eshcar Meisel, Benaya Rozen-Zvi

**Affiliations:** 1 Department of Nephrology and Hypertension, Rabin Medical Center, Petah Tikva, Israel; 2 Gray Faculty of Medical and Health Sciences, Tel- Aviv University, Tel-Aviv, Israel; 3 Department of Transplantation, Rabin Medical Center, Petah Tikva, Israel

**Keywords:** all-cause mortality, chronic kidney disease, diabetes mellitus, diabetic kidney disease, dihydropyridine calcium channel blockers, hypertension, SGLT2 inhibitors

## Abstract

**Rationale & Objective:**

Optimal antihypertensive therapy with renoprotective effects is essential to slow diabetic kidney disease progression. We evaluated the impact of dihydropyridine calcium channel blockers on kidney outcomes in patients with type 2 diabetes receiving guideline-directed renin angiotensin system inhibitors and sodium/glucose cotransporter 2 inhibitors.

**Study Design, Participants, & Settings:**

A retrospective cohort study using Clalit Health Services (2016-2021).

**Exposure:**

Adults with type 2 diabetes treated with both renin angiotensin system inhibitors and sodium/glucose cotransporter 2 inhibitors were categorized into dihydropyridine calcium channel blockers group (renin angiotensin system inhibitors + dihydropyridine calcium channel blockers, with or without other agents) or a dihydropyridine calcium channel blocker–free group (renin angiotensin system inhibitors ± other antihypertensives excluding dihydropyridine calcium channel blockers).

**Outcomes:**

The primary outcome was a major adverse kidney event (MAKE; defined as ≥40% estimated glomerular filtration rate decline or progression to kidney failure). Secondary outcomes included initiation of renal replacement therapy and all-cause mortality. Safety outcomes included hospitalizations and acute kidney injury.

**Analytical Approach:**

Inverse probability of treatment weighting was applied to balance baseline characteristics.

**Results:**

We included 31,031 patients with type 2 diabetes treated with both sodium/glucose cotransporter 2 inhibitors and renin angiotensin system inhibitors. A total of 12,172 (60.8%) received dihydropyridine calcium channel blockers, and 18,859 (39.2%) received dihydropyridine calcium channel blocker–free therapy. Median follow-up was 1,260 days. Overall, 482 patients experienced MAKE, and 2,064 patients died. Dihydropyridine calcium channel blocker use was associated with a higher risk of MAKE compared with dihydropyridine calcium channel blocker-free therapy (weighted HR, 1.33; 95% CI, 1.03-1.73; *P* = 0.03), which remained significant after accounting for competing risk of death (HR, 1.39; 95% CI, 1.13-1.7; *P* = 0.002). Dihydropyridine calcium channel blocker use was not significantly associated with all-cause mortality or safety outcomes.

**Limitations:**

The observational design is subject to residual confounding and did not allow for full characterization of medication exposure.

**Conclusions:**

In patients with type 2 diabetes treated with renin angiotensin system inhibitors and sodium/glucose cotransporter 2 inhibitors, concomitant therapy with dihydropyridine calcium channel blockers was associated with a higher risk of adverse kidney outcomes. These findings may help inform clinicians when weighing the potential risks and benefits of second-line antihypertensive therapy in this population.

## Plain-Language Summary

We studied 31,031 adults with type 2 diabetes taking both renin angiotensin system inhibitors and sodium/glucose cotransporter 2 inhibitors. We compared those also using dihydropyridine calcium channel blockers with those using other blood pressure medicines. Over a median follow-up of 3.5 years, major adverse kidney events (kidney failure or a sustained ≥40% drop in estimated glomerular filtration rate) were uncommon but occurred more often in the dihydropyridine calcium channel blockers group. After adjusting for baseline differences, dihydropyridine calcium channel blocker use was associated with a 33% higher risk of major adverse kidney events but not with a higher risk of overall mortality. In conclusion, adding a dihydropyridine calcium channel blocker to renin angiotensin system inhibitor and sodium/glucose cotransporter 2 inhibitor therapy may be associated with worse kidney outcomes, and these findings may help clinicians weigh the risks and benefits of second-line blood pressure treatment in this population.

## Introduction

Diabetic kidney disease (DKD) is the leading cause of chronic kidney disease (CKD) and incident kidney failure worldwide, affecting 20%-40% of individuals with diabetes and contributing substantially to morbidity and mortality.^1^ Hypertension, which frequently coexists with diabetes, both accelerates and results from DKD progression, making optimal antihypertensive therapy with renoprotective effects critical to clinical management.^2^

The 2021 Kidney Disease: Improving Global Outcomes (KDIGO) Guidelines recommend a renin angiotensin system inhibitors (RASis), either angiotensin-converting enzyme inhibitor (ACEi) or angiotensin receptor blocker (ARB), as first-line antihypertensive therapy in patients with diabetes and hypertension.^3^ However, evidence guiding the choice of second-line agents is limited. Guidelines often suggest either a dihydropyridine calcium channel blocker (DCCB) or a thiazide diuretic, based largely on extrapolated or nonrenal outcomes. In clinical practice, DCCBs are frequently preferred, as they effectively lower blood pressure and require minimal laboratory monitoring, unlike thiazides, which may cause electrolyte disturbances and creatinine fluctuations.^4^

Nevertheless, DCCBs exert class-specific hemodynamic effects that may be detrimental to kidney health. By selectively dilating the afferent arteriole, classic L-type DCCBs (eg, amlodipine) increase renal plasma flow and intraglomerular pressure, potentially exacerbating hyperfiltration, albuminuria, and glomerulosclerosis, pathophysiological hallmarks of DKD.^2^^,^^4^ This adverse mechanism might be offset by concomitant RASi therapy, which dilates the efferent arteriole and reduces interglomerular pressure. In a recent target trial emulation, Blum et al^4^ demonstrated that DCCBx use without RASis was associated with kidney disease progression, whereas the combination of DCCBs and RASis appeared to attenuate this risk, although replication is limited.

The emergence of sodium/glucose cotransporter 2 inhibitors (SGLT2is) has further transformed the therapeutic landscape. SGLT2is reduce glomerular hyperfiltration, slow DKD progression, and improve cardiovascular outcomes in patients with diabetes.^5^ However, the impact of DCCB treatment, when added to concomitant RASi and SGLT2i therapy, on DKD progression remains unknown.

To address this gap, we conducted a large, real-world cohort study to evaluate the association between DCCB use and kidney disease progression among patients with type 2 diabetes treated with RASi and SGLT2i.

## Research Design and Methods

### Study Population and Design

We conducted a retrospective cohort study using the electronic repositories of Clalit Health Services, the largest health care provider in Israel. A detailed description of these data sources has been published previously.^6^

We included adult (aged ≥21 years) with type 2 diabetes who received both an SGLT2i and a RASi (either ACEi or ARB) between 2016 and 2021. Patients were categorized into 2 groups: (1) the DCCB group, defined as those treated with a DCCB in combination with RASi (with or without additional antihypertensives); and (2) the DCCB-free group, comprising those treated with RASi alone or with other antihypertensive agents excluding DCCB. To evaluate whether background SGLT2i therapy modified the association, we examined a comparator cohort of patients treated with RASi and a dipeptidyl peptidase-4 inhibitor (DPP-4i) rather than an SGLT2i.

Exclusion criteria included advanced CKD at baseline (estimated glomerular filtration rate [eGFR] <15 mL/min/1.73 m^2^), dialysis treatment, prior kidney transplantation, or missing key covariates required for inverse probability of treatment weighting (IPTW).

Demographic, clinical, laboratory, and prescription data were obtained from electronic health records. Baseline covariates included age, sex, socioeconomic status (based on area of residence),^7^ time since hypertension diagnosis, blood pressure measured at the clinic, body mass index, baseline eGFR (CKD-EPI [Chronic Kidney Disease Epidemiology Collaboration] 2021 equation), urinary albumin-creatinine ratio (<30, 30-300, >300 mg/g, or missing value), baseline hemoglobin, hemoglobin A_1c_ (categorized in 1% increments with a separate group for missing values), as well as comorbid conditions and concomitant medications. We also accounted for RASi dose intensity. High-dose ACEi therapy was defined as ramipril >2.5 mg or enalapril dose >10 mg and high-dose ARB therapy as valsartan >80 mg, candesartan >8 mg, or losartan dose >50 mg. Notably, nondihydropyridine calcium channel blockers were not included among antihypertensive medications, as their use in Israel is limited and primarily indicated for rate control rather than blood pressure management. The index date was defined as the date of SGLT2i initiation, and treatment exposure was ascertained at that time point, based on medication prescriptions documented in electronic medical records.

### Outcomes

The primary outcome was a major adverse kidney event (MAKE), defined as ≥40% decline in eGFR from baseline (persisting for >3 months) or progression to kidney failure, defined as eGFR <15 mL/min/1.73 m^2^ or initiation of renal replacement therapy [RRT]).^8^

Secondary outcomes included initiation of RRT as an individual component and all-cause mortality. Safety outcomes included all-cause hospitalizations within 1 year and acute kidney injury, defined as a >50% increase in serum creatinine between 2 laboratory measurements obtained <3 months apart. Additionally, gout events were evaluated as a falsification outcome. Follow-up extended for up to 5 years from index date.

### Statistical Analysis

#### Main Analysis

Baseline characteristics were compared using *t*-test or Mann-Whitney *U* test for continuous variables, and χ^2^ test for categorical variables. IPTW was applied to adjust baseline imbalances, with propensity scores derived from multivariable logistic regression including all covariates. Covariate balance was assessed using standardized mean differences <0.1.

To assess the potential impact of residual confounding, we calculated E-values for the main analyses.

Weighted cause-specific Cox proportional hazards models were used to estimate hazard ratios (HRs) and 95% confidence intervals (CIs) for the association between DCCB use and outcomes. Weight adjusted competing risk analyses (Fine–Gray models) were performed to account for death as a competing event. Proportional hazards assumptions were assessed using Schoenfeld residuals and log(–log[S]) plots.

Survival outcomes were visualized using weighted Kaplan–Meier curves, with log-rank tests employed for group comparisons.

To support the robustness of our findings, we preformed an additional analysis restricted to patients treated with either DCCB or hydrochlorothiazide (HCTZ) as the comparator. Individuals receiving both agents were classified as the DCCB group. Thiazide diuretics were selected as an active comparative given their role as an alternate recommended second-line option for hypertension management.

To account for blood pressure control during follow-up, we further adjusted the main analyses for time-varying hypertension status, defined using ≥2 blood pressure measurements obtained during the first year after the index date.

#### Subgroup Analysis

Prespecified subgroup analyses were conducted using interaction terms between DCCB use and the following variables: age (>65 vs ≤65 years), sex, eGFR (≥60 vs <60 mL/min/1.73 m^2^), body mass index (<30 vs ≥30 kg/m^2^), proteinuria (urinary albumin-creatinine ratio; <30 vs ≥30 mg/g), and ischemic heart disease.

#### Sensitivity Analyses

To evaluate the robustness of findings, we conducted several sensitivity analyses. First, we restricted the cohort to patients with complete baseline proteinuria data. Second, we limited the analysis to patients receiving ≥1 additional antihypertensive agent beyond RASi. Finally, we repeated the analysis without adjustment for proteinuria, given the potential effects of DCCB on albumin exertion.

#### Supporting Analyses

To evaluate whether the use of SGLT2i modified the association of DCCB with outcomes, we compared patients treated with SGLT2i with those treated with DPP-4i. In addition, we assessed outcomes according DCCB subtype (amlodipine vs new-generation DCCB such as lercanidipine) and dosage, classifying high-dose therapy as amlodipine 10 mg/d or lercanidipine 20 mg/d, and lower doses as low-dose therapy.

### Ethics

The study protocol was reviewed and approved by the local ethics committee of the Institutional Review Board of Rabin Medical Center, Israel (approval number RMC-0133-22) and conducted in accordance with the Declaration of Helsinki. Informed consent was waived due to the retrospective design. The study followed Strengthening the Reporting of Observational Studies in Epidemiology reporting guidelines.

## Results

### Baseline Characteristics

Between 2016 and 2021, 31,031 patients with type 2 diabetes treated with both SGLT2i and RASi were included. Of these, 12,172 (39.2%) received a DCCB (DCCB group), and 18,859 (60.8%) patients received a DCCB-free antihypertensive regimen (DCCB-free group).

Before IPTW, patients receiving DCCBs were older, more often female, had a higher socioeconomic status, and had a longer duration since hypertension diagnosis. They also had higher systolic blood pressure and body mass index, lower eGFR, hemoglobin, and hemoglobin A_1c_ levels, and a higher prevalence of albuminuria and heavy albuminuria. Hypertension was more common in the DCCB group. Concomitant therapy differed as well: DCCB users more frequently received ARBs (60.6%) than ACEis and were more often treated with high-dose ACEis (29.8%) or ARBs (33.7%), whereas the DCCB-free group was more commonly treated with ACEis (64.8), and fewer were treated with high-dose ACEis (29.3%) or ARBs (7.6%). In addition, HCTZ use was more frequent among DCCB users (33.7% vs 22.2%), who were also more commonly prescribed β-blockers and α-blockers and less commonly prescribed furosemide (Table 1).

**Table 1:** Characteristics of the Study Population by Treatment Strategy Before and After Inverse Probability Treatment Weighting

Variable	Crude Cohort				IPTW				
	DCCB-free	DCCB	*P*-value	SMD	All	DCCB-free	DCCB	*P*-value	SMD
N	18,859	12,172			**31,031**				
Demographic characteristics									
Age, y (mean ± SD)	66.14 ± 10	68.95 ± 9.2	< 0.001	0.29		67.43 ± 9.78	67.55 ± 9.54	0.27	0.01
Female, n (%)	7,155 (37.7)	4,850 (39.8)	< 0.001	0.43		39.51	39.19	< 0.001	< 0.001
SES (mean ± SD)	3.14 ± 1.13	3.26 ± 1.12	< 0.001	0.11		3.19 ± 1.12	3.2 ± 1.14	0.5	0.01
Time since HTN diagnosis, y (mean ± SD)	9.57 ± 6.42	13.39 ± 4.89	< 0.001	0.68		11.15 ± 6.12	11.41 ± 6.06	< 0.001	0.04
Physical examinations									
Systolic BP, mmHg (mean ± SD)	131 ± 15	135 ± 15	< 0.001	0.28		133 ± 15	133 ± 15	0.04	0.02
Diastolic BP, mmHg (mean ± SD)	74.35 ± 9.68	74.45 ± 10.07	0.367	0.01		75 ± 10	75 ± 10	0.02	0.03
1-y systolic BP, mmHg (mean ± SD)	129 ± 14	133 ± 15	< 0.001			130 ± 15	132 ± 14	< 0.001	0.11
1-y diastolic BP, mmHg (mean ± SD)	73 ± 9.5	74 ± 10	0.004			73 ± 10	74 ± 10	< 0.001	0.05
BMI, kg/m^2^ (mean ± SD)	30.56 ± 5.57	31.05 ± 5.35	< 0.001	0.9		30.86 ± 5.67	30.98 ± 5.44	0.08	0.02
Laboratory examinations									
eGFR, mL/min/1.73 m^2^ (mean ± SD)	87.37 ± 19.78	83.07 ± 19.72	< 0.001	0.22		85.32 ± 20.02	85.03 ± 19.8	0.2	0.01
HbA1C, % (mean ± SD)	8.19 ± 1.53	8.02 ± 1.41	< 0.001	0.12		8.11 ± 1.49	8.11 ± 1.44	0.65	0.01
Missing HbA1C, n (%)	1,619 (8.58)	971 (7.98)	0.06	0.01		8.19	8.43	0.44	0.01
Hb level (mean ± SD)	13.73 ± 1.66	13.57 ± 1.62	< 0.001	0.1		13.64 ± 1.67	13.63 ± 1.63	0.8	0.001
Missing prot (%)	1,241 (6.58)	677 (5.56)	< 0.001	0.04		6.2	6.27	0.8	0.003
Any albuminuria^a^(%)	7,720 (40.94)	6,555 (53.85)	< 0.001	0.26		46.3	45.46	0.8	0.01
Heavy albuminuria (%)	1,784 (9.46)	2,126 (17.47)	< 0.001	0.24		13.2	12.74	0.11	0.01
Comorbidities									
HTN(%)	15,988 (84.8)	11,991 (98.5)	< 0.001	0.46		90.37	91.48	< 0.001	0.04
IHD (%)	6,388 (33.9)	3,849 (31.6)	< 0.001	0.05		32.91	32.04	0.1	0.02
CHF (%)	601 (3.2)	341 (2.8)	< 0.001	0.02		2.9	2.86	0.47	0.01
Atrial fibrillation	1,514 (11.47)	2,164 (12.43)	0.01	0.03		11.98	11.96	0.95	0
COPD (%)	2,691 (14.3)	1,631 (13.4)	0.03	0.03		14.02	14.08	0.03	0.002
Stroke (%)	1,082 (5.7)	880 (7.2)	< 0.001	0.06		6.6	6.55	0.86	0.002
PHTN (%)	624 (3.3)	396 (3.3)	0.82	< 0.001		3.38	3.28	0.62	0.01
Hypothyroidism (%)	2,237 (11.9)	1,570 (12.9)	0.01	0.03		12.49	12.57	0.83	0.002
Medications									
ACEI, n (%)	12,220 (64.8)	5,110 (42)	< 0.001	0.46		54.5	54.17	0.56	0.01
High dose ACEI^b^, n (%)	5,529 (29.3)	3,628 (29.8)	0.36	0.01		28.85	29.79	0.07	0.02
ARB, n (%)	6,844 (36.3)	7,380 (60.6)	< 0.001	0.49		47.26	47.62	0.52	0.01
High dose ARB^c^, n (%)	1,440 (7.6)	4,099 (33.7)	< 0.001	0.68		19.76	18.74	0.02	0.03
HCTZ, n (%)	4,180 (22.2)	4,099 (33.7)	< 0.001	0.26		29.97	29.15	0.11	0.02
Furosemide, n (%)	2,090 (11.1)	1,265 (10.40)	0.06	0.23		10.94	11.01	0.83	0.002
BBs, n (%)	9,630 (51.1)	7,039 (60.0)	< 0 .001	0.18		55.06	54.11	0.09	0.02
Alpha blockers (%)	525 (2.8)	1,284 (10.5)	< 0.001	0.33		7.21	6.17	< 0.001	0.04
MRA (%)	1,405 (7.5)	785 (6.4)	0.001	0.04		7.01	6.77	0.4	0.01
Metformin (%)	1,4967 (79.4)	9,694 (79.6)	0.56	0.01		79.63	79.47	0.74	0.004
Insulin (%)	5,932 (31.5)	3,679 (30.2)	0.02	0.03		31.28	31.12	0.02	0.003
GLP1 agonists (%)	3,126 (16.6)	2,131 (17.5)	0.03	0.02		17.2	17.16	0.03	0.002
Sulphonylurea (%)	2,753 (14.6)	1,811 (4.9)	0.51	0.01		14.86	15.05	0.64	0.01

*Note:* Continuous variables are presented as mean ± standard deviation and categorical variables as n (%). Variables with absolute standardized differences after propensity score weighting of <0.1.

Abbreviations: ACEi, angiotensin-converting enzyme inhibitor; ARB, angiotensin receptor blocker; BB, β-blocker; BP, blood pressure; BMI, body mass index; COPD, chronic obstructive pulmonary disorder; DCCB, dihydropyridine calcium channel blocker; eGFR, estimated glomerular filtration rate; GLP1, glucagon like peptide-1; Hb, hemoglobin; HCTZ, hydrochlorothiazide; HTN, hypertension; HF, heart failure; IHD, ischemic heart disease, IPTW, inverse probability treatment weighting; MRA, mineralocorticoid receptor blocker; PHTN, pulmonary hypertension; SD, standard deviation; SES, socioeconomic status; SMD, standardized mean difference.

a Albuminuria was calculated from spot albumin-creatinine ratios (normal: 0-29 mg/g; any albuminuria >30 mg/g; heavy albuminuria >300 mg/g).

b High dose of ACEi was defined as: ramipril dose >2.5 mg, enalapril dose >10 mg.

c High dose of ARB was defined as: valsartan dose >8 mg, candesartan dose >8 mg, losartan dose >50 mg.

**Figure 1:**
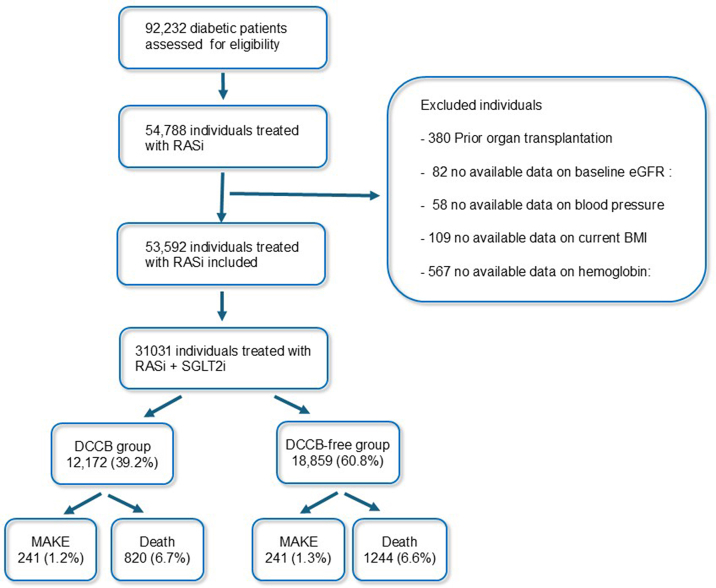
Flow chart of study. Major adverse kidney event (MAKE) is defined as ≥40% decline in estimated glomerular filtration rate (eGFR) from baseline or progression to kidney failure (eGFR <15 mL/min/1.73 m^2^ or initiation of renal replacement therapy). BMI, body mass index; DCCB, dihydropyridine calcium channel blocker; RASi, renin angiotensin system inhibitor; SGLT2i, sodium/glucose cotransporter 2 inhibitor.

After IPTW, baseline covariates were well balanced (standardized mean difference <0.1). The weighted cohort had a mean age of 67 years, 61% were male, and mean eGFR was 85 mL/min/1.73 m^2^ (Table 1). Median follow-up was 1,260 days (1,225 in the DCCB group and 1,280 in the DCCB-free group). Figure 1 summarizes the patient breakdown.

### Kidney Outcomes and Mortality During Follow-up

During follow-up, 482 patients developed MAKE, yielding an incidence rate of 4.8 events per 1,000 patient-years (95% CI, 4.3-5.2). This included 299 patients with a sustained ≥40% decline in eGFR, 40 who progressed to eGFR <15 mL/min/1.73 m^2^ and 143 who initiated RRT. In total, 2,064 patients died, corresponding to 20.3 deaths per 1,000 patient-years (95% CI, 19.4-21.2). In the DCCB group, the event rates for MAKE, RRT initiation, and death were 6.2 (95% CI, 5.4-7.0), 1.8 (95% CI, 1.4-2.3), and 20.9 (95% CI, 19.5-22.4) per 1,000 patient-years, respectively. The corresponding rates in the DCCB-free group were 3.9 (95% CI, 3.4-4.4), 1.2 (95% CI, 0.9-1.5), and 19.9 (95% CI, 18.8-21.0) per 1,000 patient-years, respectively (Fig 1).

MAKE risk was higher in the DCCB group than in the DCCB-free group in both crude (HR, 1.63; 95% CI, 1.37-1.95; *P* < 0.001) and IPTW-weighted analyses (HR, 1.33; 95% CI, 1.03-1.73; *P* = 0.03). This association persisted in competing risk models accounting for death (HR, 1.39; 95% CI, 1.13-1.7; *P* = 0.002), for which the E-value was 1.51, and remained significant after additional adjustment for time-varying hypertension status (HR, 1.36; 95% CI, 1.11-1.67; *P* = 0.003 without competing risk adjustment and HR, 1.36; 95% CI, 1.13-1.67; *P* = 0.003 with competing-risk adjustment).

The risk of RRT initiation was higher in the DCCB group than in the DCCB-free group in crude analyses, both before and after accounting for death as a competing risk (HR, 1.54; 95% CI, 1.11-2.14; *P* = 0.01 and HR, 1.52; 95% CI, 1.1-2.1; *P* = 0.01, respectively). In the IPTW-weighted analyses, this association attenuated and showed only a trend toward significance in competing risk models (HR, 1.42; 95% CI, 0.97-2.08; *P* = 0.07). No significant association between DCCB use and all-cause mortality was observed in either crude or IPTW-weighted analyses (Table 2).

**Table 2:** Crude and Weighted Hazard Ratios for Kidney Outcomes and Mortality Associated With DCCB Versus DCCB-Free Therapy

N=31,031	Covariate	No. of events			Crude HR		Weighted^a^ HR		Adjusted-weighted^b^ HR	
		All	DCCB-free n=18,859	DCCB n=12,172	HR (95% CI)	*P*-value	HR (95% CI)	*P*-value	HR (95% CI)	*P*-value
Efficacy outcomes	Composite kidney outcome (MAKE)	482	241	241	1.63 (1.37-1.95)	< 0.001	1.33 (1.03-1.73)	0.03	1.33 (1.03-1.72)	0.03
	MAKE with competing risk^c^ adjustment				1.63 (1.36-1.94)	< 0.001	1.39 (1.13-1.7)	0.002	1.36 (1.11-1.67)	0.003
	RRT	143	74	69	1.54 (1.11-2.14)	0.01	1.48 (0.89-2.48)	0.3		
	RRT with competing risk adjustment				1.52 (1.1-2.1)	0.01	1.42 (0.97-2.08)	0.07		
	All-cause mortality	2,064	1,244	820	1.06 (0.97-1.2)	0.22	0.92 (0.83-1.03)	0.15		
Safety outcomes	Hospitalization^d^	8,319	5,018	3,301	1.02 (0.98-1.07)	0.39	1.02 (0.98-1.07)	0.39		
	AKI	1,955	1,148	807	1.12 (1.03-1.23)	0.01	1.06 (0.95-1.2)	0.3		
	Gout	1,034	558	476	1.36 (1.2-1.53)	1.53	0.99 (0.84-1.17)	0.9		

*Note:* Major adverse kidney event (MAKE) is defined as ≥40% decline in eGFR from baseline or progression to kidney failure (eGFR <15 mL/min/1.73 m^2^ or initiation of RRT).

Abbreviations: AKI, acute kidney injury; CI, confidence interval; DCCB, dihydropyridine calcium channel blocker; eGFR, estimated glomerular filtration rate; HR, hazard ratio; RRT, renal replacement therapy.

a Inverse probability of treatment weighting (IPTW) was applied with propensity scores derived from multivariable logistic regression including all covariates. Weights were stabilized, winsorized at 5 standard deviations, and covariate balance assessed using standardized mean differences (SMD <0.1). Weighted for age, sex, socioeconomic status, time from hypertension diagnosis, body mass index, systolic blood pressure, diastolic blood pressure, hemoglobin A_1c_, eGFR, urinary albumin-creatinine ratio; history of ischemic heart disease, heart failure, atrial fibrillation, cerebrovascular disease, chronic obstructive pulmonary disorder, pulmonary hypertension, hypothyroidism; renin angiotensin system inhibitor type and dose; use of hydrochlorothiazide, furosemide, β-blockers, α-blockers, mineralocorticoid receptor antagonist, metformin, sulfonylurea, insulin, glucagon-like peptide 1 receptor agonist.

b Adjusted weighted HR additionally adjusted for time-varying hypertension status, defined using ≥2 blood pressure measurements during the first year.

c Adjustment for competing risk of death.

d All-cause hospitalizations within 1 year.

**Figure 2:**
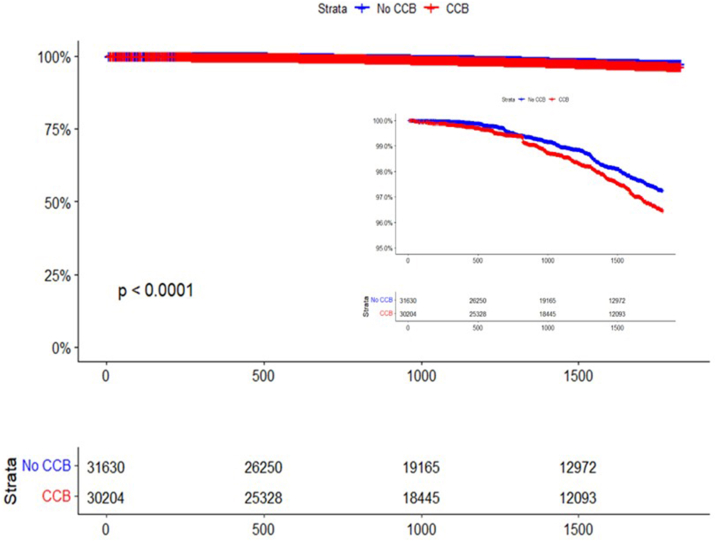
Weighted cumulative incidence curves for major adverse kidney events (MAKEs) according to treatment. Cumulative incidence curves were estimated with the Aalen-Johansen estimator accounting for competing risks between MAKE and all-cause mortality. MAKE defined as ≥40% decline in estimated glomerular filtration rate (eGFR) from baseline or progression to kidney failure (eGFR <15 mL/min/1.73 m^2^ or initiation of renal replacement therapy). Cumulative incidence curves were weighted for age, sex, socioeconomic status, body mass index, systolic blood pressure, diastolic blood pressure, hemoglobin A_1c_, eGFR, urinary albumin-creatinine ratio; history of ischemic heart disease, heart failure, cerebrovascular disease, chronic obstructive pulmonary disorder, pulmonary hypertension, hypothyroidism; use of hydrochlorothiazide, β-blockers, α-blockers, vasodilators, mineralocorticoid receptor antagonist, metformin, sulfonylurea, insulin, glucagon-like peptide 1 receptor agonist. Abbreviation: CCB, calcium channel blocker.

**Figure 3:**
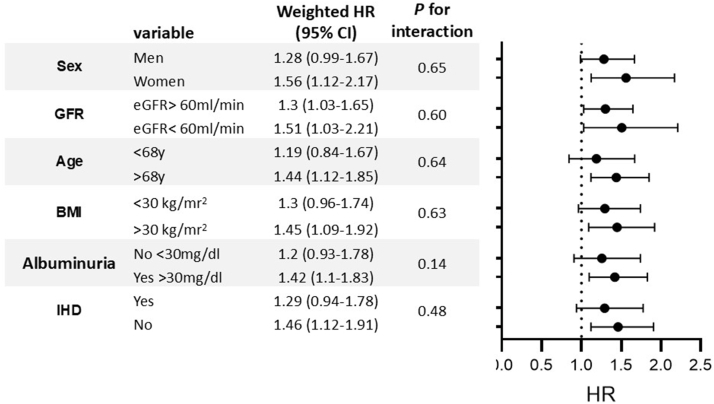
Subgroup analyses: weighted hazard ratios (HRs) for the association between dihydropyridine calcium channel blocker use and major adverse kidney events. Weighted for age, sex, socioeconomic status, body mass index (BMI), systolic blood pressure, diastolic blood pressure, hemoglobin A_1c_, estimated glomerular filtration rate (eGFR), urinary albumin-creatinine ratio; history of ischemic heart disease, heart failure, cerebrovascular disease, chronic obstructive pulmonary disorder, pulmonary hypertension, hypothyroidism; use of hydrochlorothiazide, β-blockers, α-blockers, vasodilators, mineralocorticoid receptor antagonist, metformin, sulfonylurea, insulin, glucagon-like peptide 1 receptor agonist. Abbreviations: CI, confidence interval; IHD, ischemic heart disease.

Kaplan–Meier analysis confirmed a higher cumulative incidence of MAKE among DCCB users (*P* < 0.0001) (Fig 2). Subgroup analyses showed no significant interaction across subgroups defined by age, sex, body mass index, CKD stage, albuminuria, or ischemic heart disease (Fig 3).

### Active-Comparator Analysis: DCCB versus HCTZ

In the active-comparator analysis restricted to patients receiving either DCCB or HCTZ monotherapy (n = 15,100), 286 patients developed MAKE, yielding an incidence rate of 5.4 events per 1,000 patient-years (95% CI, 4.8-6.1). This included 184 patients with a sustained ≥40% decline in eGFR, 14 who progressed to eGFR <15 mL/min/1.73 m^2^ and 88 who initiated RRT. During follow-up, 1,061 patients died, corresponding to 20.0 deaths per 1,000 patient-years (95% CI, 18.8-21.3). In the DCCB group, event rates for MAKE, RRT initiation, and all-cause mortality were 6.2 (95% CI, 5.4-7.0), 1.8 (95% CI, 1.4-2.3), and 21.0 (95% CI, 19.6-22.5) per 1,000 patient-years, respectively, compared with 3.4 (95% CI, 2.5-4.6), 1.3 (95% CI, 0.8-2.1), and 17.3 (95% CI, 15.2-19.6) per 1,000 patient-years in the HCTZ group

MAKE risk was higher with DCCBs than with HCTZ in the crude analysis, both before (HR, 1.86; 95% CI, 1.37-2.53; *P* < 0.001) and after accounting for death as a competing risk (HR, 1.83; 95% CI, 1.35-2.5; *P* < 0.001). In IPTW-weighted analyses, the associations showed a trend toward significance (HR, 1.37; 95% CI, 0.98-1.92; *P* = 0.06) and became significant in competing risk models (HR, 1.43; 95% CI, 1.04-1.97; *P* = 0.03; E-value, 1.24). The association remained significant after additional adjustment for time-varying hypertension status (weighted HR, 1.45; 95% CI, 1.04-2.00; *P* = 0.03, competing risk adjusted). In both crude and IPTW-weighted analyses, DCCB use showed no significant association with RRT initiation or all-cause mortality (Table 3).

**Table 3:** Crude and Weighted Hazard Ratios for Kidney Outcomes and Mortality Associated With DCCB Versus Thiazide Therapy

Variable	Covariate	No. of Events			Crude HR		Weighted^a^ HR		Weighted^a^ adjusted^b^ HR	
N=15,100		All	DCCB- free n=3,933	DCCB n=11,167	HR (95% CI)	*P*-value	HR (95% CI)	*P*-value	HR (95% CI)	*P*-value
Efficacy outcomes	Composite kidney outcome (MAKE)	286	49	237	1.86 (1.37-2.53)	< 0.001	1.37 (0.98-1.92)	0.06	1.34 (0.96-1.88)	0.08
	MAKE with competing risk adjustment^c^				1.83 (1.35-2.5)	< 0.001	1.43 (1.04-1.97)	0.03	1.45 (1.04-2.0)	0.03
	RRT	88	19	69	1.32 (0.79-2.2)	0.29	0.98 (0.57-1.71)	0.96		
	RRT with competing risk adjustment				1.32 (0.79-2.2)	0.29	1.03 (0.61-1.75)	0.91		
	All-cause mortality	1,061	247	814	1.23 (1.07-1.42)	0	0.95 (0.81-1.12)	0.56		
Safety outcomes	Hospitalization^d^	4,288	1,044	3,244	1.1 (1.03-1.18)	0.007	0.99 (0.91-1.07)	0.75		
	AKI	1,042	245	797	1.22 (1.05-1.4)	0.008	1.03 (0.88-1.2)	0.74		
	Gout	616	144	472	1.19 (0.99-1.44)	0.06	0.86 (0.7-1.06)	0.16		

*Note:* Major adverse kidney event (MAKE) is defined as ≥40% decline in eGFR from baseline or progression to kidney failure (eGFR <15 mL/min/1.73 m^2^ or initiation of RRT).

Abbreviations: AKI, acute kidney injury; CI, confidence interval; DCCB, dihydropyridine calcium channel blocker; eGFR, estimated glomerular filtration rate; HR, hazard ratio; RRT, renal replacement therapy.

a Weighted for age, sex, socioeconomic status, time from hypertension diagnosis, body mass index, systolic blood pressure, diastolic blood pressure, hemoglobin A_1c_, eGFR, urinary albumin-creatinine ratio; history of ischemic heart disease, heart failure, atrial fibrillation, cerebrovascular disease, chronic obstructive pulmonary disorder, pulmonary hypertension, hypothyroidism; renin angiotensin system inhibitor type and dose; use of hydrochlorothiazide, furosemide, β-blockers, α-blockers, mineralocorticoid receptor antagonist, metformin, sulfonylurea, insulin, glucagon-like peptide 1 receptor agonist.

b Adjusted weighted HR additionally adjusted for time-varying hypertension status, defined using ≥2 blood pressure measurements obtained during the first year.

c Adjustment for competing risk of death.

d All-cause hospitalizations within 1 year.

### Safety Outcomes

Overall, 8,319 all-cause hospitalizations (26.8%) occurred within the first year, along with 1,955 acute kidney injury events (6.3%) and 1,034 gout events (3.3%). No significant between-group differences were observed for hospitalizations, acute kidney injury, or gout (Table 2).

### Sensitivity Analysis

When restricted to patients with complete albuminuria data (n = 27,113), 461 MAKEs and 1,883 deaths were recorded. DCCB users had increased MAKE risk (weighted HR, 1.35; 95% CI, 1.01-1.81; *P* = 0.04), remaining consistent after competing risk adjustment (HR, 1.37; 95% CI, 1.11-1.69; *P* = 0.004), without mortality difference.

In an analysis restricted to patients receiving ≥2 antihypertensives (n = 22,352), 424 MAKEs and 1,758 deaths occurred. A trend toward higher MAKE was observed (weighted HR, 1.25; 95% CI, 0.97-1.62; *P* = 0.09), becoming significant after competing risk adjustment (HR, 1.32; 95% CI, 1.06-1.64; *P* = 0.001). No mortality difference was observed.

Finally, in the analysis performed without adjustment for proteinuria, MAKE risk remained higher in the DCCB group (weighted HR, 1.5; 95% CI, 1.18-1.9; *P* < 0.001), remaining consistent after competing risk adjustment (HR, 1.54; 95% CI, 1.25-1.89; *P* < 0.001), with no mortality difference (Table 4).

**Table 4:** Crude and Weighted Hazard Ratios From Sensitivity Analyses for Kidney Outcomes and Mortality Associated With DCCB Versus Non-DCCB Therapy

Covariates	No of events			Crude		Weighted^a^ HR	
	All	DCCB-free	DCCB	HR (95% CI)	*P*-value	HR (95% CI)	*P*-value
Complete albuminuria data (n=27,113)							
Composite kidney outcome (MAKE)	461	232	229	1.61 (1.34-1.93)	< 0.001	1.35 (1.01-1.81)	0.04
MAKE with competing risk adjustment^b^				1.6 (1.33-1.92)	< 0.001	1.37 (1.11-1.69)	0.004
All-cause mortality	1,883	1,128	755	1.07 (0.98-1.18)	0.14	0.93 (0.83-1.04)	0.22
Patients receiving ≥ 2 antihypertensive medications (n=22,352)							
Composite kidney outcome (MAKE)	424	183	241	1.33 (1.1-1.61)	0.004	1.25 (0.97-1.62)	0.09
MAKE with competing risk adjustment				1.34 (1.11-1.62)	0.003	1.32 (1.06-1.64)	0.01
All-cause mortality	1,758	938	820	0.88 (0.8-0.96)	0.005	0.95 (0.85-1.07)	0.39
Sensitivity analysis: unadjusted for proteinuria (n=31,031)							
Composite kidney outcome (MAKE)	482	241	241	1.63 (1.37-1.95)	< 0.001	1.5 (1.18-1.9)	< 0.001
MAKE with competing risk adjustment				1.63 (1.36-1.9)	< 0.001	1.54 (1.25-1.89)	< 0.001
All-cause mortality	2,064	1,244	820	1.06 (0.97-1.2)	0.22	0.98 (0.88-1.09)	0.68

*Note:* Major adverse kidney event (MAKE) is defined as ≥40% decline in eGFR from baseline or progression to kidney failure (eGFR <15 mL/min/1.73 m^2^ or initiation of renal replacement therapy).

Abbreviations: DCCB, dihydropyridine calcium channel blocker; eGFR, estimated HR, hazard ratio.

a Weighted for age, sex, socioeconomic status, time from hypertension diagnosis, body mass index, systolic blood pressure, diastolic blood pressure, hemoglobin A_1c_, eGFR, urinary albumin-creatinine ratio; history of ischemic heart disease, heart failure, atrial fibrillation, cerebrovascular disease, chronic obstructive pulmonary disorder, pulmonary hypertension, hypothyroidism, renin angiotensin system inhibitor type and dose; use of hydrochlorothiazide, furosemide, β-blockers, α-blockers, mineralocorticoid receptor antagonist, metformin, sulfonylurea, insulin, glucagon-like peptide 1 receptor agonist.

b Adjustment for competing risk of death.

### DCCB Dosage and Subtype

Among DCCB users, 8,568 (72.4%) were treated with low doses, and 3,273 (27.6%) were treated with high doses. No interaction was observed between kidney outcomes and DCCB dose (high vs. low, *P* for interaction = 0.53). Amlodipine was the most common subtype (n = 7,384; 62.4%), and lercanidipine was used in 4,219 (35.6%) patients. Analysis according to subtype showed no interaction (amlodipine vs lercanidipine, *P* for interaction = 0.71) (Table S1 and Table S2).

### SGLT2i versus DPP-4i Comparisons

Among patients treated with RASi and DPP-4i (n = 22,561), 1,504 MAKEs (6.7%) and 4,252 deaths (18.8%) occurred (Fig S1). In both crude and IPTW-weighted analyses, concomitant DCCB use was associated with a higher risk of MAKE compared with DCCB-free regimen (weighted HR, 1.18; 95% CI, 1.04-1.33, *P* = 0.009), and the association persisted in the competing risk model accounting for death (weighted HR, 1.23; 95% CI, 1.1-1.37; *P* < 0.001). No significant difference in all-cause mortality was observed.

Interaction testing showed no evidence that SGLT2is use modified the association between DCCB therapy and CKD progression (*P* for interaction = 0.43) (Table S3).

## Discussion

In this large, real-world cohort of patients with type 2 diabetes receiving guideline-directed therapy with RASi and SGLT2i, concomitant use of DCCB was associated with a higher risk of kidney outcomes, with no significant differences in all-cause mortality or other safety outcomes. The association was consistent across prespecified subgroups, multiple sensitivity analyses, and a competing risk model accounting for death, supporting the robustness of the observed signal.

Hypertension is both a cause and a consequence of DKD, and optimizing antihypertensive therapy is essential to slowing progression. Although RASis are established first-line agents, second-line selection is less well defined. DCCBs are widely used because they are effective and well tolerated. However, classic L-type DCCBs preferentially dilate the afferent arteriole, potentially increasing intraglomerular pressure and hyperfiltration, a key driver of DKD progression.^4^^,^^9^, ^10^, ^11^

This mechanism is supported by experimental work in 5/6-nephrectomized rat model, where DCCB worsened glomerulosclerosis compared with non-dihydropyridine CCBs or ACEi.^10^^,^^12^ In contrast, ACEis reduce intraglomerular pressure and hyperfiltration via efferent arteriolar dilatation and have consistently demonstrated nephroprotection in both experimental models and clinical trials.^13^, ^14^, ^15^, ^16^

Clinical evidence has been mixed. In the Avoiding Cardiovascular Events Through COMbination Therapy in Patients Living with Systolic Hypertension (ACCOMPLISH) trial, ACEi + DCCB reduced renal and cardiovascular events compared with ACEi + HCTZ, although few participants had albuminuria.^17^^,^^18^ In contrast, a small, randomized trial in diabetic patients with albuminuria showed greater albuminuria reduction with ACEi + thiazide compared with ACEi + DCCB.^19^ More recently, Blum et al^4^ reported higher albuminuria and kidney failure risk with DCCB use without RASi, with attenuation when combined with renin angiotensin system blockade, although their cohort included relatively few patients with diabetes.

Our study extends prior literature by focusing on patients with type 2 diabetes, receiving both RASi and SGLT2i, the current standard of care targeting hyperfiltration, and still observed worse kidney outcomes with concomitant DCCB use. In diabetes, the potential deleterious effect of DCCBs on kidney disease progression may be more pronounced. Hyperglycemia increases proximal sodium reabsorption (via sodium/glucose cotransporter 2 and sodium/glucose cotransporter 1 upregulation), reduces distal sodium delivery to the macula densa, and triggers maladaptive afferent vasodilation, intraglomerular hypertension, and hyperfiltration. SGLT2is partially mitigate these changes by restoring distal sodium delivery and reducing glomerular hyperfiltration.^11^^,^^20^ Despite this optimized regimen of SGLT2i in combination with ACEi, in the present study, DCCB exposure remained associated with higher kidney risk compared with DCCB-free regimens, persisting after competing adjustment for death and additional adjustment for time-varying hypertension status, without evidence of effect modification across subgroups. The secondary endpoint of RRT initiation showed only a trend toward significance in fully adjusted analyses, likely reflecting limited power given the relatively small number of kidney failure events in this intensively treated cohorts.

Findings were supported in an active-comparative analyses directly comparing DCCBs with HCTZ, a guideline-endorsed second-line option. Although the restricted cohort was smaller and IPTW estimates showed only a trend, the associations became significant in fully adjusted competing-risk models and remained significant after accounting for time-varying hypertension status. Importantly, the modest increase in MAKE in both the main and active-comparator cohorts was driven primarily by sustained eGFR decline. To minimize misclassification from hemodynamic dips, we required a repeatedly confirmed ≥40% decline in eGFR, intended to capture a more durable loss of kidney function. Consistency in the thiazide-based comparative cohort, in which early hemodynamic eGFR changes may also occur, support interpreting this sustained ≥40% decline as clinically meaningful kidney function loss.

The observed association appeared confined to kidney outcomes, with no excess risk of mortality, hospitalizations, or acute kidney injury in either the main or active-comparator analyses, aligning with prior work suggesting predominantly renal rather than systemic, adverse signals associated with DCCB use.^1^^,^^2^^,^^18^

Sensitivity analyses further reinforced consistency: the association persisted in patients receiving ≥2 antihypertensive agents and among those with complete proteinuria data and appeared stronger when proteinuria adjustment was omitted, compatible with a pathway in which DCCBs influence hyperfiltration and albuminuria.

Taken together, these results raise concern that, despite their widespread use, DCCBs may not represent the optimal antihypertensive strategy for patients with diabetes at risk for CKD, even in the setting of contemporary kidney-protective therapy. Notably, our findings are consistent with those of a recent target trial emulation in patients with advanced CKD (eGFR <30 mL/min/1.73 m^2^), showing better kidney outcomes with diuretics + RASi than with DCCB + RASi,^2^ supporting the concern that DCCB-associated arteriolar effects may be unfavorable in high-risk CKD populations with persistent glomerular hyperfiltration.

We initially hypothesized that DCCB-associated hyperfiltration might blunt SGLT2i nephroprotection. However, DCCB use was associated with higher kidney risk in both SGLT2i and DPP-4i background-therapy cohorts, with no interaction with SGLT2i use, suggesting the association is largely independent of SGLT2i therapy.

The substantially lower MAKE risk in the SGLT2i cohort compared with the DPP-4i cohort is consistent with established SGLT2i nephroprotection. Notably, the higher event rate in the DPP-4i cohort also increased statistical power to detect the association with DCCB exposure. Nevertheless, this between-class comparison remains exploratory and susceptible to treatment selection and residual confounding

Although newer-generation DCCBs such as lercanidipine may affect additional calcium-channel subtypes and theoretically mitigate hyperfiltration and proteinuria,^9^^,^^10^ we did not detect a difference by DCCB subtype or dose. Although our retrospective design may have limited our ability to detect dose- or subtype-specific interactions, notably, our results are consistent with prior studies that have not shown renal superiority of newer-generation DCCBs.^21^

Strengths of this study include the large community-based cohort, contemporary background therapy with both RASi and SGLT2i, and robust analytic approaches including IPTW and competing risk models. We also acknowledge limitations. First, as with any observational study, residual confounding and residual bias due to nonrandom missingness cannot be fully excluded. Second, given our aim to evaluate long-standing antihypertensive therapy in the context of more recently adopted SGLT2is, we were unable to determine the precise date of the antihypertensive treatment initiation. Third, the retrospective design of the study limited our ability to assess fully achieved blood pressure levels and trajectories during follow-up, medication adherence, treatment duration, or subsequent modification in therapy; therefore, the potential for exposure misclassification and time-dependent confounding cannot be excluded. Finally, generalizability should be interpreted cautiously, as our cohort is predominantly White; therefore, applicability to more ethnically diverse populations and other health care settings is uncertain. Although we have no a priori reason to expect effect differences by race/ethnicity, variation in baseline CKD risk, comorbid condition burden, social determinants of health, and medication access/prescribing patterns could modify observed associations, and validation in more diverse cohorts is warranted.

In conclusion, among patients with type 2 diabetes receiving RASi and SGLT2is, concomitant therapy with DCCBs was consistently associated with an increased risk of CKD progression. Given the observational nature of the study, these findings should be interpreted cautiously but may help inform clinical decision making when weighing the risks and benefits of second-line antihypertensive therapy in this high-risk population.
