# Supplementary material for: Dihydropyridine Calcium Channel Blocker Therapy and Risk of CKD Progression in Type 2 Diabetes Treated With Renin Angiotensin System Inhibitors and SGLT2 Inhibitors: A Real-World Retrospective Cohort Study
Source: Kidney Med. 2026 May 8;8(7):101394. doi: 10.1016/j.xkme.2026.101394 (PMC13311150; doi:10.1016/j.xkme.2026.101394)
Supplement: Supplementary File (PDF) — Figure S1; Table S1-S3 [file mmc1.pdf]

Supplementary Material

Table S1- Interaction analysis of DCCB dosage and the association of DCCB therapy with kidney outcomes

Table S2- Interaction analysis of DCCB subtype and the association of DCCB therapy with kidney outcomes

Table S3- Interaction analysis of SGLT2 inhibitors versus DPP-4 inhibitors use and DCCB-associated risk with kidney outcomes

Figure S1- Flow chart of study – Full cohort

	Low dose				High dose				
**Covariate**	**N**	**No of events**	**Weighted[&] HR (95% CI)**	***P*-value**	**N**	**No of events**	**Weighted[&] HR (95% CI)**	***P*-value**	***P* for interaction**
Composite kidney outcome (MAKE)	8568	416	1.39 (1.06-1.83)	0.02	3273	307	1.35 (0.85-2.16)	0.21	0.53
MAKE with competing risk adjustment[#]			1.39 (1.12-1.72)	0.03			1.36 (0.96-1.93)	0.08	
All-cause mortality		1868	0.94 (0.84-1.06)	0.32		1440	0.99 (0.78-1.25)	0.91	

Table S1- Interaction analysis of DCCB dosage and the association of DCCB therapy with kidney outcomes

Major adverse kidney event (MAKE), defined as ≥40% decline in eGFR from baseline or progression to kidney failure (eGFR <15 mL/min/1.73m² or initiation of renal replacement therapy).

High dose of DCCB was defined as: Amlodipine dose >5mg, Lercanidipines dose>10mg

[#]Adjustment for competing risk of death

[&]Weighted for age, sex, Socioeconomic status, time from HTN diagnosis, BMI, SBP, DBP, hemoglobin HbA1C, estimated glomerular filtration rate (eGFR), urinary albumin-to-creatinine ratio (ACR), history of ischemic heart disease, heart failure,atrial fibrillation, cerebrovascular disease, COPD,PHTN, hypothyroidism, RASI kind and dose, HCTZ, furosemide, b-blockers, a-blockers, MRA, metformin, sulfonylurea, insulin, GLP-1

	Amlodipine				Lercandipine				
**Covariate**	**N**	**No of events**	**Weighted& HR (95% CI)**	***P*-value**	**N**	**No of events**	**Weighted& HR (95% CI)**	***P*-value**	***P* for interaction**
Composite kidney outcome (MAKE)	7384	376	1.32 (0.99-1.75)	0.06	4219	344	1.47 (0.98-2.2)	0.06	0.71
MAKE with competing risk adjustment#			1.39 (1.13-1.7)	0.002			1.47 (1.14-1.89)	0.003	
All-cause mortality		1693	0.94 (0.81-1.08)	0.37		1594	0.91 (0.79-1.06)	0.23	

Table S2- Interaction analysis of DCCB subtype and the association of DCCB therapy with kidney outcomes

Major adverse kidney event (MAKE), defined as ≥40% decline in eGFR from baseline or progression to kidney failure (eGFR <15 mL/min/1.73m² or initiation of renal replacement therapy).

#Adjustment for competing risk of death

&Weighted for age, sex, Socioeconomic status, time from HTN diagnosis, BMI, SBP, DBP, hemoglobin HbA1C, estimated glomerular filtration rate (eGFR), urinary albumin-to-creatinine ratio (ACR), history of ischemic heart disease, heart failure,atrial fibrillation, cerebrovascular disease, COPD,PHTN, hypothyroidism, RASI kind and dose, HCTZ, furosemide, b-blockers, a-blockers, MRA, metformin, sulfonylurea, insulin, GLP-1

Covariates	No of events			Crude		Weighted[&] HR		*P* for interaction
	All	DCCB-free	DCCB	HR	*P*-value	HR	*P*-value	
**The full cohort - patients treated with RASi and either SGLT2 inhibitors or DPP4 inhibitors**								0.43
**n=**	53592	31881	21711					
Composite kidney outcome (MAKE)	1986 (3.7%)	913 (2.9%)	1073 (4.9%)	1.8 (1.65-1.97)	<0.001	1.23 (1.1-1.38)	<0.001	
MAKE with competing risk adjustment[#]				1.69 (1.59-1.8)	<0.001	1.29 (1.17-1.43)	<0.001	
All-cause mortality	6316 (11.8%)	3597 (11.3%)	2719 (12.5%)	1.14 (1.09-1.2)	<0.001	0.94 (0.89-1.0)	0.05	
**Patients treated with RASi and DPP4 inhibitors**								
**n=**	22561	13022	9539					
Composite kidney outcome (MAKE)	1504 (6.7%)	672 (5.2%)	832 (8.7%)	1.78 (1.6-1.97)	<0.001	1.18 (1.04-1.33)	0.009	
MAKE with competing risk adjustment[#]				1.76 (1.59-1.94)	<0.001	1.23 (1.1-1.37)	<0.001	
All-cause mortality	4252 (18.8%)	2353 (18.1%)	1899 (19.9%)	1.13 (1.06-1.2)	<0.001	0.94 (0.88-1.01)	0.10	

Table S3- Interaction analysis of SGLT2 inhibitors versus DPP-4 inhibitors use and DCCB-associated risk of CKD progression

Major adverse kidney event (MAKE), defined as ≥40% decline in eGFR from baseline or progression to kidney failure (eGFR <15 mL/min/1.73m² or initiation of renal replacement therapy).

[#]Adjustment for competing risk of death

[&]Weighted for age, sex, Socioeconomic status, time from HTN diagnosis, BMI, SBP, DBP, hemoglobin HbA1C, estimated glomerular filtration rate (eGFR), urinary albumin-to-creatinine ratio (ACR), history of ischemic heart disease, heart failure,atrial fibrillation, cerebrovascular disease, COPD,PHTN, hypothyroidism, RASI kind and dose, HCTZ, furosemide, b-blockers, a-blockers, MRA, metformin, sulfonylurea, insulin, GLP-1

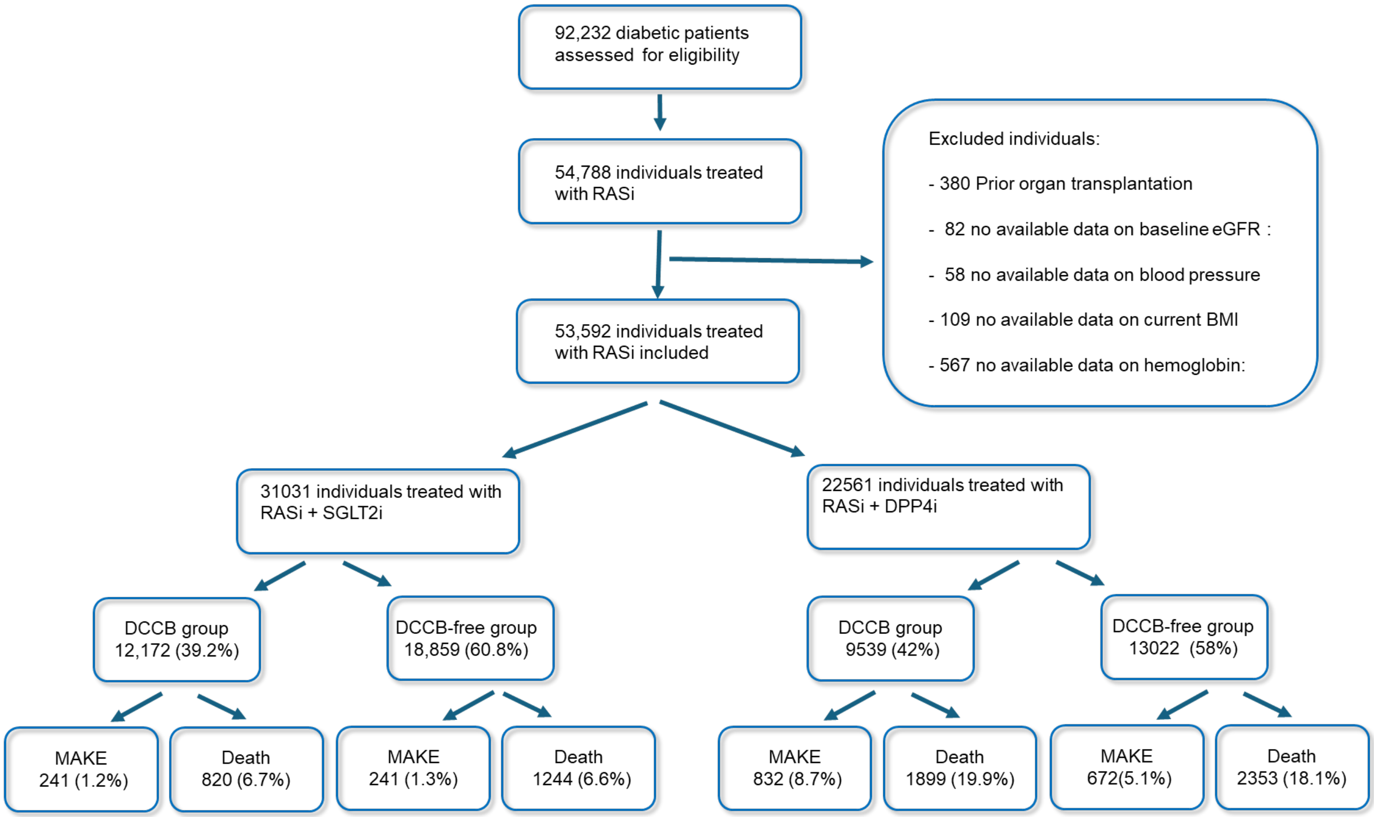

Figure S1- Flow chart of study – Full cohort
Major adverse kidney event (MAKE), defined as ≥40% decline in eGFR from baseline or progression kidney failure (eGFR <15 mL/min/1.73m² or initiation of renal replacement therapy- RRT
